# Knee Pads Do Not Affect Physical Performance in Young Female Volleyball Players

**DOI:** 10.3390/children8090748

**Published:** 2021-08-30

**Authors:** Anja Lazić, Milovan Bratić, Stevan Stamenković, Slobodan Andrašić, Nenad Stojiljković, Nebojša Trajković

**Affiliations:** 1 Faculty of Sport and Physical Education, University of Niš, 18000 Niš, Serbia; anja.lazic96@hotmail.com (A.L.); bratic@fsfv.ni.ac.rs (M.B.); sstamenkovic.fsfv@gmail.com (S.S.); snesadif@yahoo.com (N.S.); 2 Faculty of Economics, University of Novi Sad, 24000 Subotica, Serbia; slobodan.andrasic@ef.uns.ac.rs

**Keywords:** jumping performance, speed, agility, team sport

## Abstract

Knee pads have become increasingly popular among volleyball players. Given the fact high-intensity activities that are crucial to successfully playing this sport lead to an increased risk of a knee injury, the primary use of knee pads is to prevent potential injury. However, no research has been carried out to explain the effects of knee pads on the most important physical abilities in volleyball players, thus directly affecting performance. This study was undertaken to determine the effects of knee pads on the explosive power of the lower extremities, linear speed, and agility in young female volleyball players. In two separated sessions, 84 female volleyball players (age: 14.83 ± 0.72 years; height: 163.19 ± 8.38 cm; body mass: 53.64 ± 10.42 kg; VE: 5.30 ± 3.39 years) completed squat jumps (SJ), countermovement jumps (CMJ) with and without arm swing, linear sprints at 5-m and 10-m, modified *t*-test, and 5-10-5 shuttle test. Data analyses included descriptive statistics, paired sample *T*-tests and use of effect size (ES). There was no statistical difference between the two conditions for SJ (*p* = 0.156; ES = 0.18), CMJ (*p* = 0.817; ES = 0.03), CMJ with arm swing (*p* = 0.194; ES = 0.14), linear sprint at 5 m (*p* = 0.789; ES = 0.03) and 10 m (*p* = 0.907; ES = −0.01), modified *t*-test (*p* = 0.284; ES = 0.13), and 5-10-5 shuttle test (*p* = 0.144; ES = 0.19). Wearing knee pads has neither an inhibitory nor positive effects on explosive power of the lower extremities, linear speed, and agility in young female volleyball players.

## 1. Introduction

During a volleyball match, 80% of the points obtained are the result of high-intensity activities performed with maximal and submaximal intensity [1]. On average, volleyball players perform 250–300 high-intensity activities [2], over 100 jumps [3], and a large number of sprints up to 10 m [3], which together with technical and tactical elements [4] define volleyball as an intermittent and complex team sport, where high-intensity movements are followed by a short period of low-intensity activities [5,6]. Also, frequent changes in the rules of the game [6], the popularization and increasing in professionalism [7], specific dimensions of the court [8], and thus the high demands of the game [9], have led to a high level of physical fitness—primarily explosive power of the upper and lower extremities, speed, and agility represented as a crucial factor for successfully playing this sport at elite level [10,11,12]. Specifically, explosive power is manifested through a wide range of volleyball elements—ball hitting, jumps, and blocks [4,7], while speed and agility are manifested through sudden changes of movement direction [10,11,12], where maximal speed rarely develops [13], and movements are closely related to the unpredictable nature of actions [14]. The frequency and importance of these abilities lead to the fact that volleyball players are more prone to knee injuries, particularly to a ruptured anterior cruciate ligament [15,16]. Landings are often performed on one or both legs [17] and are associated with multiplanar cutting and pivoting movements that may increase stress load on the knee joint [14], especially in female volleyball players [18].

Prevention of potential volleyball injuries has received a lot of research attention [19]. Knee pads have become increasingly popular in volleyball players, being designed to give dynamic stability and prevent potential injury [20]. While there is no research on knee pads, knee braces have been extensively researched in rehabilitation and prevention of knee injuries in athletes [20,21,22,23,24,25,26,27,28,29]. The purpose of these braces is to effectively reduce knee valgus during lateral forces [30] without limitations in athletic performance. However, available studies addressing knee braces in injury prevention show contradictory results. While some studies have reported positive effects of knee braces in injury prevention [23,24], others have shown no effect [27,28,29], and Deppen with colleagues [25] together with Grace and colleagues [26], have even reported an increase in knee injury rates. Because of that, the role of knee braces is still poorly understood. This has an additional impact on athletes, who mostly avoid wearing this type of brace because of the potential negative impact on athletic performance [20,31,32,33]. Within this area of investigation, a number of studies [30,34,35,36,37,38,39,40,41] have reported conflicting results. Improvements in physical performance have been shown in [34,35] in contrast to those who have shown negative effects [36,40] and no effects [30,39,40,41] of wearing knee braces on physical performance.

However, most of these studies have utilized healthy or injured athletes, recreational athletes or non-athletes, this resulting in that the performed tests do not show the real nature of a specific competitive sport, especially in volleyball where knee pads are worn constantly and where the use of knee pads may result in decreased performance when participants become accustomed to wearing them. Since knee pads are worn by many volleyball players, it is critical to determine if wearing them significantly affects physical performance. The aim of this study was to determine the effects of knee pads on the explosive power of the lower extremities, linear speed, and agility in young female volleyball players.

## 2. Materials and Methods

### 2.1. Participants

A total of 84 young female volleyball players (age: 14.83 ± 0.72 years; height: 163.19 ± 8.38 cm; body mass: 53.64 ± 10.42 kg) volunteered to participate in the study. All participants were members of the same volleyball club. Participants had 5.30 ± 3.39 years of volleyball experience. Participants were free of injuries and medical conditions that might have placed them at risk for safe participation in the study. All participants were informed of the study procedures and provided written informed consent prior to participation, including parental consent for participants under 18 years of age.

### 2.2. Procedures

A repeated-measures study design was used in this study. Each participant completed two experimental trials on an indoor, hardwood volleyball court under similar environmental conditions at the same time of the day (19:00–21:00). Experimental trials were separated by 72 h. The first trial session involved testing without knee pads. After 72 h the same procedure was repeated with the use of knee pads. Before both experimental trials, participants performed a standardized warm-up, consisting of moderate-intensity jogging (5–10 min) and static and dynamic stretching (5 min) without knee pads during the first session and with knee pads during the second session. The study was conducted in accordance with the Novi Sad University Human Research Ethics Committee guidelines (ethical approval number: 20/2019; approval date of Ethic Committee approval: 20 October 2020).

### 2.3. Tests

#### 2.3.1. Vertical Jump Assessment

Three tests were used to assess the explosive power of the lower extremities: squat jump (SJ), countermovement jump (CMJ), and countermovement jump with arm swing. Participants performed each jump three times. The break between each repetition of the jump was 30 s, while the break between series of new jumps was 5 min. Each participant was instructed to jump naturally and as high as they could, performing all jumps with maximal effort. The highest value of the vertical jump height for all three groups of tests was included in the statistical analysis. Optojump (Optojump, Microgate, Bolzano, Italy) was used to estimate the vertical height of the jump, and its validity and reliability were confirmed, considering low coefficients of variation (2.7%) and low random errors (+2.81 cm) [42]. The participants performed three jumps with arm swing and were instructed to jump naturally and as high as they could, performing all jumps with maximal effort.

#### 2.3.2. Linear Speed Assessment

The linear sprinting speed was evaluated at 5 m and 10 m using a photocell system (Witty, System, Microgate, Bolzano, Italy). Each participant repeated the test three times with at least 2 min of rest between trials, and the fastest time was recorded for further statistical analysis. Photocells were placed at a distance of 5 m and 10 m from the starting line. Photocells were placed 0.4 m above the ground with an accuracy of 0.001 m/s to minimize the effect of hand swing when passing through the gate [43].

#### 2.3.3. Agility Assessment

Agility assessment was performed using two tests—modified *t*-test and 5-10-5 shuttle test. Participants repeated each of test three times, with a break of 30 s between attempts and a 5 min break between the tests. The fastest time on agility tests was taken in further statistical analysis of the data. Modified *t*-test procedures were in accordance with Sassi et al. [44]. Upon command of the examiner, the participant sprinted towards the cone B set at the distance of 5 m and touched the base of the cone with the right hand. Then, they turned left and shuffled sideways to cone C (2.5 m), touching the base with the left hand. Then, shuffling sideways to the cone D (5 m), touching its base with the right hand, followed up by shuffle to the left to the cone B (5 m), touching its base with the left hand and running back to the cone A (5 m). The time was stopped when the start with photocell was passed by. Trials were deemed unsuccessful if participants failed to touch a designated cone, crossed their legs while shuffling, or were unable to face forward at all times. The 5-10-5 shuttle test was performed according to National Strength and Conditioning Association (NSCA) protocols [45] and measured with photo cell timing gates (Brower Timing Systems, Draper, UT). Briefly, three cones were placed five yards apart on the surface of the volleyball court. The each participant started in a three point stance and upon command sprinted right and touched the line at the first cone with his hand, the subject then turned 180 degrees and sprinted to touch the left cone line with his hand, then turned 180 degrees to sprint back to the start. The 5-10-5 shuttle run has been shown to be a reliable (ICC = 0.90, SEM = 0.12) test of change of direction [46].

### 2.4. Statistical Analysis

Statistical analysis was performed by the IBM SPSS statistics program (version 26.0; Inc., Chicago, IL, USA). Descriptive statistics were used to obtain basic information about the participants. The normality of data distribution was determined by Kolmogorov–Smirnov test for all dependent variables, while to determine the differences in explosive power of the lower extremities, linear speed and agility between the two conditions, the paired sample *T*-tests were used. The magnitude of difference between the two conditions was measured with effect size (ES) analyses and interpreted as: trivial ≤ 0.20; small = 0.2–0.49; moderate = 0.50–0.79; large ≥ 0.80 [47]. Data are presented as mean ± standard deviation (SD) and statistical significance level was set at *p* < 0.05

## 3. Results

Descriptive statistics of participants are shown in Table 1 while means and standard deviations for each dependent variable in the non-braced and braced condition are shown in Table 2. ES (effect size) comparisons between the conditions are presented in Table 2.

Table 1 shows that the mean age of the 84 female volleyball players was 14.83 ± 0.72 years (range: 12–20 years), with a mean height of 163.19 ± 8.38 cm (range: 147.0–179.5 cm) and mean weight 53.64 ± 10.42 kg (range: 35.0–85.7 kg); mean volleyball experience was 5.30 ± 3.39 years (range: 1–15 years).

The Kolmogorov–Smirnov tests showed that data were normally distributed and homogeneity of variance was confirmed using the Levene’s test. Paired sample *T*-tests were used to determine potential differences between braced and non-braced conditions in height of vertical jumping, linear sprinting speed, and agility. Table 2 shows non-significant differences found for any variable between the two conditions. Wearing knee pads resulted in a trivial, non-significant increase in SJ (ES = 0.18), CMJ (ES = 0.03) and non-significant increase in CMJ with arm swing (ES = 0.14); trivial, non-significant reduction in the time required to perform linear sprint at 5 m (ES = 0.03); and trivial, non-significant reduction in the time required to perform linear sprint at 10 m (ES = −0.01). Finally, wearing knee pads resulted in trivial, non-significant increase in the time required to perform modified *t*-test (ES = 0.13) and 5-10-5 shuttle test (ES = 0.19).

## 4. Discussion

The aim of this study was to determine the effects of wearing knee pads on young female volleyball players, on the explosive power of the lower extremities, linear speed, and agility. The results showed that there were no statistically significant differences between the two conditions and that wearing knee pads did not improve, but also did not inhibit specific volleyball performance.

Although a considerable body of research has been done on the effects of knee bracing in injured athletes [35,48,49] and non-athletes [30,39], less attention has been paid to the population of interest [41,50,51]. Past studies in non-injured populations have yielded some important insights into the effects of wearing a knee brace on height during vertical jumping [30,39,41,50,51]. Taken altogether, the data presented here provide evidence that the braces do not affect vertical jump height. Studies of Batlaci et al. [30] and Veldhuizen et al. [39] investigated vertical jump height in young, uninjured participants and found no statistically significant differences between braced and non – braced conditions. However, due to the sample of participants [30,39] as well as the selection of the tests for assessing the explosive power of the lower extremities [30], these findings do not aplly to athletes. Substantially, studies involving athletes [41,50] have not defined definite differences between the two conditions. Rishiraj and colleagues [50] conducted five testing sessions in each condition. Statistically significant differences were noticeable at the initial testing session, but at the final testing session, no difference was found in the vertical jump. Mortaza and colleagues [41] included 31 male athletes in one testing session and found no effect of bracing on vertical jump. While we could not find initial significant effects of bracing in volleyball players, in braced and non-braced condition, Rishiraj et al. [50] and Veldhuizen et al. [39] found initial, acute decrease in vertical jump height. A possible explanation could be given by Aktas and Baltaci [51], who emphasize that the external load exerted by the knee braces can apply excessive pressure to the skin and change the physiology by inhibiting the mechanoreceptors of the knee, which may results in negative acute effects of knee bracing. However, there is no reliable evidence that this decrease is due to wearing knee braces, given that after the period of 14 h [50] and 28 days [39], there were no statistically significant differences between braced and non—braced conditions. Also, in this study, the period between the trials was 72 h, it should be added that volleyball players use knee pads more and longer than other athletes, so we should not rule out potential earlier adaptation to wearing a pad as a mediator of non—significant differences.

In contrast to the findings of this study, where sprint performance at 5 m and 10 m did not differ between braced and non-braced conditions, previous studies [39,50,52,53,54] have demonstrated that knee braces inhibit running velocity at short distances. These findings are less surprising if we consider the sample of participants. Veldhuzen et al. [39] included eight healthy volunteers and found that sprinting time during 60 m Dash test was 4% longer than in non-braced volunteers. Similarly, Rishiraj et al. [50] reported longer time at 10 m sprint. However, both studies concluded that after getting accustomed to knee brace, after 4 weeks [39] and 14 h [50], results came back to baseline compared to unbraced condition. In other studies, participants were rugby players [52], young male athletes [50], and young college athletes in unspecified sports [53]. These findings are not generalizable to volleyball players. A possible reason for this discrepancy might be that sprint performance depends on the type of knee brace. In a study conducted by Green and colleagues [55], the effects of six different knee braces on speed and agility were analyzed. Four out of six braces negatively affected athletic performane. This finding is congruent with the work of Albright et al. [54], which showed that longer time required to complete sprint tests depends on a variety of factors, among which the most important are the weight and design of the knee brace. The weight of the knee brace leads to altered activity of the knee extensors and hip flexors [53], which are the most active muscles while jogging, running or sprinting [56]. However, a more relevant study to explain the results obtained is a study of Stephens [57] who reported that knee bracing had no effect on sprint speed. This is a statement with a strong background, because basketball and volleyball have many more similarities as team, indoor sports with a specific manifestation of physical fitness [58] than other sports presented in the disscusion.

Wearing a knee brace may negatively affect agility and athletic performance [59], taking into account the fact that agility is a crucial ability in many sports. However, there was no statistical difference between the two conditions in time required to complete the agility tests with or without knee pads. A limited number of studies [55,59,60] have addressed the fact that agility maneuvers are not affected by knee bracing. However, while the former study of Rishiraj et al. [60] showed no differences between the two conditions in agility slalom test and the figure-of-eight test, the latter study, which included 27 young male athletes [50], resulted in initially longer time required to complete agility test in braced-group, but after 14 h, results came back to baseline as in unbraced condition. They attributed decrease in performance to the adaptation to wearing a knee brace. The weakness of this research is that they examined agility without cutting movements, in regard to a straight line. Similarly, Green et al. [55] showed that agility performance depends on the type of knee brace, where significant differences were found only with one of six braces.

These findings are less surprising if we consider that the volleyball players were well-rested during the assessment, without the previous high-intensity activities, so it would be more relevant to determine the impact of knee pads on vertical jump, speed, and agility in condition of acute fatigue, similar to that during a volleyball match. A possible reason for this discrepancy might be that non-significant differences are probably a consequence of the weight and design of volleyball knee pads, which are lighter, with different designs, in relation to the majority of knee braces presented in the discussion. At present our dataset is limited to studies which do not address knee pads or a specific volleyball population. Therefore, these findings are not sufficient to determine whether knee pads inhibit or improve volleyball performance. Future studies are to be carried out to explore effects of specific knee pads in volleyball players, taking into account their wide use in volleyball.

## 5. Conclusions

Pointing to the majority of volleyball players who use knee pads, this research was conducted with the aim of determining the effect of knee pads on important factors of volleyball performance. The conclusion is that wearing knee pads has neither an inhibitory nor a positive effect on the explosive power of the lower extremities; linear speed; and agility in young female volleyball players. However, to our knowledge, this is the first study that tried to examine the effects of knee pads in athletes and the first study that involved a specific population of volleyball players. Considering the importance of high level of these abilities for successful volleyball performance and at the same time the wide use of knee pads in volleyball players, further research in this area of is needed. This study provides data that will help coaches and volleyball players in resolving doubts about the use of knee pads for players of all ages, especially for young players whose locomotor system has not yet been formed. The findings of this study highlight that wearing knee pads do not deteriorate the physical performance of volleyball players and that their further use is desirable for the primary purpose, which is the prevention of potential knee injuries and consequences of frequent falls, which all together may have a positive impact on the psychological status and overall performance of the athlete. Moreover this study supports researchers who will try to examine and explain the impact of knee pads and to what extent they affect the prevention of potential knee injuries in volleyball players.

## Figures and Tables

**Table 1:** Descriptive statistics of participants.

Outcome Measure (Unit)	N	Min	Max	Mean ± SD
Age (year)	84	12	20	14.83 ± 0.72
VE (year)	84	1	15	5.30 ± 3.39
Height (cm)	84	147.0	179.5	163.19 ± 8.38
Body mass (kg)	84	35.0	85.7	53.64 ± 10.42

VE—volleyball experience; N—a total number of participants; SD—standard deviation.

**Table 2:** Differences and effect size values between the conditions.

Outcome Measure	Condition		*p* Value	ES
	Without Knee Pads	With Knee Pads		
**Vertical jump height**				
SJ (cm)	23.55 ± 5.03	24.47 ± 5.06	0.156	0.18—trivial
CMJ without arm swing (cm)	24.46 ± 5.46	24.61 ± 5.56	0.817	0.03—trivial
CMJ with arm swing (cm)	29.91 ± 6.40	30.90 ± 7.30	0.194	0.14—trivial
**Linear sprint speed**				
5 m—sprint (s)	1.25 ± 0.15	1.25 ± 0.12	0.789	0.03—trivial
10 m—sprint (s)	2.18 ± 0.18	2.17 ± 0.17	0.907	−0.01—trivial
**Agility**				
modified *t*-test (s)	7.40 ± 0.58	7.48 ± 0.64	0.284	0.13—trivial
5-10-5 test (s)	5.96 ± 0.35	6.03 ± 0.40	0.144	0.19—trivial

SJ—squat jump; CMJ—counter movement jump; *p* value– significant difference, *p* = 0.05; ES—effect size.
